# Effects of resveratrol on *in vitro* circadian clock gene expression in young and older human adipose-derived progenitor cells

**DOI:** 10.18632/aging.205292

**Published:** 2024-01-06

**Authors:** Sophie G.C. Kapar, Maria F. Pino, Fanchao Yi, Miguel A. Gutierrez-Monreal, Karyn A. Esser, Lauren M. Sparks, Melissa L. Erickson

**Affiliations:** 1 Translational Research Institute, AdventHealth, Orlando, FL 32804, USA; 2 Department of Physiology and Aging, University of Florida, Gainesville, FL 32610, USA

**Keywords:** circadian clock, circadian rhythm, aging, adipose-derived progenitor cells, resveratrol

## Abstract

Observational studies in preclinical models demonstrate age-related declines in circadian functions. We hypothesized that age would be associated with declines in function of cell-autonomous circadian clocks in human tissue. Accordingly, we cultured adipose progenitor cells (APCs) from previously collected white-adipose tissue biopsies from abdominal subcutaneous depots of young (Age: 23.4 ± 2.1 yrs) vs. older female participants (Age: 70.6 ± 5.9 yrs). Using an *in vitro* model, we compared rhythmic gene expression profiles of core clock components, as an indicator of circadian oscillatory function. We observed consistent circadian rhythmicity of core clock components in young and older-APCs. Expression analysis showed increased levels of some components in older-APCs (CLOCK, CRY1, NR1D1) vs. young. We also investigated resveratrol (RSV), a well-known longevity-enhancing effector, for its effects on rhythmic clock gene expression profiles. We found that RSV resulted in gained rhythmicity of some components (CLOCK and CRY), loss of rhythmicity in others (PER2, CRY2), and altered some rhythmic parameters (NR1D1 and NR1D2), consistent in young and older-APCs. The observation of detectable circadian rhythmicity retained *in vitro* suggests that the oscillatory function of the cell-autonomous core clock in APCs is preserved at this stage of the aging process. RSV impacts core clock gene expression in APCs, implicating its potential as a therapeutic agent for longevity by targeting the core clock.

## INTRODUCTION

Numerous aspects of physiology, metabolism, and behavior follow 24h rhythmic cycles that are driven by a biological network of endogenous molecular clocks [1]. Observational studies in preclinical models reveal that circadian functions weaken with age [2], and this may be linked to age-related health declines. Cross-sectional studies in humans similarly demonstrate dampened circadian behavior in older adults compared to younger adults [3]. Yet still, age-related changes in endogenous molecular clocks in humans is not well characterized, and addressing this knowledge gap may unveil a new understanding of aging biology. We sought to address this by examining age-related changes in the core clock mechanism in a peripheral tissue from humans, which has not been done previously.

In brief, the core clock mechanism is a self-sustaining transcription-translation feedback loop that exists in virtually every cell [1]. Circadian function of cell-autonomous clocks is observable by assessing the rhythmic oscillatory activity of the core clock mechanism—which is retained *in vitro* [4–7]. This approach is advantageous because it gives insights into the oscillatory function of the core clock independent of any influence that may be present *in vivo*, giving precise insight into the capabilities of the core clock mechanism itself. Previous studies have used this approach to demonstrate that preadipocytes isolated from adipose tissue depots in humans maintain detectable circadian rhythms *in vitro*, evidenced by 24h rhythmic patterns of mRNA expression of core clock components [8, 9]. Informed by this work, our study aimed to investigate age-related differences in the oscillatory function of the core clock mechanism by examining whether rhythmic expression patterns are altered in preadipocytes derived from older adults relative to young adults.

Resveratrol (RSV) is a natural polyphenol that extends lifespan and healthspan in preclinical models [10–13]. These benefits are hypothesized to be mediated by SIRT1, a highly conserved nicotinamide adenine dinucleotide (NAD+)-dependent histone deacetylase [13–18] that is reduced in aging, and also interacts with the core clock mechanism [19]. Previous studies show that RSV alters the expression of core clock genes in young and old human lung fibroblasts [20]. RSV has also been shown to be useful in counteracting metabolic dysregulation induced by a high-fat diet, through altering rhythmic clock gene expression [21]. These studies and others [18, 21–23] support the hypothesis that RSV may be beneficial for aging by activating SIRT1 and interacting with the core clock. Whether RSV modifies the core clock mechanism, assessed as altering the expression patterns of core clock genes, in human adipose tissue has never been tested. Moreover, if age impacts the ability of the core clock mechanism to respond to RSV is unknown.

Thus, the primary objective of this study was to compare *in vitro* rhythmic clock gene expression in WAT-derived progenitor cells (APCs) derived from young (23.4 ± 2.1 yrs) and older (70.6 ± 5.9 yrs) female participants. We hypothesized that older-APCs would exhibit altered *in vitro* rhythmic clock gene expression relative to young-APCs. The secondary objective of this study was to determine the effects of RSV on *in vitro* rhythmic clock gene expression in these same young and older-APCs, and explore the putative role of SIRT1 activation. We hypothesized that RSV treatment impacts *in vitro* rhythmic clock gene expression patterns, by restoring circadian rhythmicity and increasing rhythmic amplitude, in both older-APCs and young-APCs.

## RESULTS

### APC donor characteristics

We assessed *in vitro* rhythmic clock gene expression in APCs that were cultured from previously collected adipose tissue biopsy specimens that were harvested from the abdominal depots of young (N=5) and older (N=5) female participants. Clinical characteristics of the APC donors are shown in Table 1. With the exception of age (*P* ≤ 0.0001) and systolic blood pressure (*P* = 0.027), there were no significant differences in race, height, weight, BMI, waist-hip ratio, DBP, fasting glucose, HbA_1C_, total cholesterol, HDL, LDL, LDL/HDL ratio, VLDL, triglycerides and non-HDL cholesterol between groups.

**Table 1:** Participant characteristics.

**Participant characteristics**	**Young**	**Older**	**P-value**
Gender (F), n	5	5	-
Race (black/white), n	1/4	1/4	>0.999
Age (y)	23.40 ± 2.07	70.60 ± 5.86	<0.0001^*^
Height (m)	1.59 ± 0.09	1.60 ± 0.08	0.852
Weight (kg)	73.83 ± 6.78	75.81 ± 20.98	0.846
BMI (kg/m^2^)	29.48 ± 5.04	29.20 ± 5.95	0.938
Waist-Hip ratio	0.844 ± 0.05	0.874 ± 0.08	0.489
Systolic Blood Pressure (mmHg)	116.70 ± 3.29	132.00 ± 12.24	0.027^*^
Diastolic Blood Pressure (mmHg)	69.90 ± 7.70	74.30 ± 6.99	0.372
Fasting glucose (mg/dL)	84.75 ± 9.18	86.80 ± 10.08	0.762
HbA_1C_ (%)	4.95 ± 0.68	5.50 ± 0.21	0.125
Total Cholesterol (mg/dL)	160.50 ± 17.02	192.40 ± 54.13	0.299
HDL (mg/dL)	63.00 ± 10.23	61.40 ± 11.19	0.831
LDL (mg/dL)	83.25 ± 26.04	113.20 ± 50.52	0.321
LDL/HDL ratio	1.35 ± 0.59	1.90 ± 0.93	0.341
VLDL (mg/dL)	14.25 ± 6.75	17.80 ± 7.98	0.502
Triglycerides (mg/dL)	71.75 ± 33.64	88.20 ± 39.59	0.530
Non-HDL Cholesterol (mg/dL)	97.50 ± 21.06	131.00 ± 56.29	0.301

Data are presented as mean ± standard deviation. All measurements were taken following an overnight fast. ^*^P <0.05 young vs. older compared with two-sample t-test. Gender and race were compared with Fisher’s Exact test. F, female; BMI, body mass index; Hb, hemoglobin; HDL, high-density lipoprotein; LDL, low-density lipoprotein; VLDL, very low-density lipoprotein. Blood work data missing for N = 1 young participant.

### *In vitro* rhythmic clock gene expression in APCs from young and older participants

**Figure 1:**
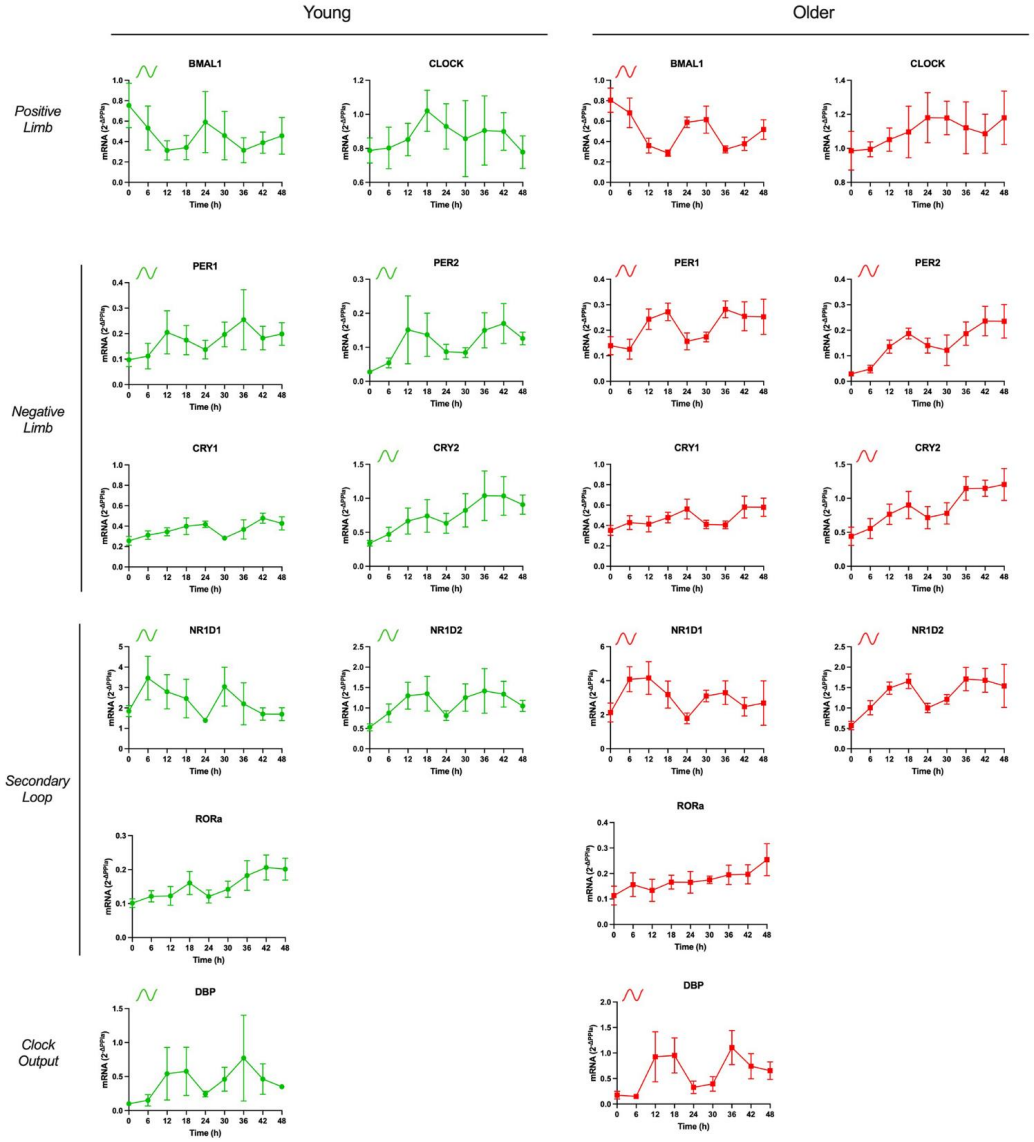
**mRNA expression of components of core clock mechanism are rhythmic in human adipose-derived progenitor cells (APCs) from young and older participants.** APCs were synchronized with 30% fetal bovine serum (FBS) for two hrs and treated with vehicle for 12 hrs. RNA was extracted from APCs, harvested every six hrs from 12 to 60 hrs post-synchronization. Abundance of transcripts of core clock gene components was quantified by RT-PCR and normalized to Peptidylprolyl isomerase A (PPIA). Data are presented as mean ± SEM. When ARSER analysis detected a significant rhythm (*P*<0.05), symbols in the graphics represent oscillation in young (green), in older (red), or in both groups. BMAL1: brain and muscle ARNT-like protein-1, CLOCK: circadian locomotor output cycles kaput; PER1/2: period 1/2, CRY1/2: cytochrome 1/2, D site of albumin promoter (albumin D-box) binding protein, RORα: RAR related orphan receptor A; NR1D1/2, nuclear receptor subfamily 1 group D member 1/2.

To assess the rhythmic pattern of core clock gene expression in APCs derived from young and older female participants, we measured mRNA levels of core clock genes including BMAL1, CLOCK, PER1/2, CRY1/2. We also measured mRNA levels of the nuclear receptors NR1D1, NR1D2, and RORα which are part of a secondary regulatory loop contributing to the core clock, as well as the ubiquitous clock output gene, DBP. For these assessments, APCs were synchronized with 30% FBS for 2h. After 12h, APCs were then harvested every 6h for 48 h and mRNA levels were assessed (Figure 1). From these time points, we tested rhythmic profiles for significant circadian rhythmicity, which determines goodness of fit to a cosine wave, using ARSER analysis [24].

In young-APCs, circadian rhythmicity was detected in core clock components BMAL1, PER1, PER2, CRY2, NR1D1 and NR1D2 and the clock output gene DBP (all *P*<0.05). In older-APCs, circadian rhythmicity was detected in these same components, BMAL1, PER1, PER2, CRY2, NR1D1 and NR1D2 as well as DBP (all *P*<0.05). Also, rhythmic parameters were compared between young vs. older-APCs. These parameters included rhythmic period (duration of one complete rhythmic cycle), phase (location in time of rhythmic peak) and amplitude (half the distance from the rhythmic peak to the trough) [24]. Older-APCs were characterized by PER2 and NR1D2 phase delay, and longer period length for DPB expression, relative to young (Table 2; all *P*<0.05).

**Table 2:** Comparison of rhythmic parameters of *in vitro* rhythmic clock gene expression profiles in human adipose-derived progenitor cells (APCs) isolated from young vs. older participants.

**Gene**	**Parameter**	**Young-APCs (N=5)**		**Older-APCs (N=5)**		***P*-value**
		**Average**	**SEM**	**Average**	**SEM**	
**BMAL1**	Period	23.5	0.94	24.1	0.21	0.57
	Phase	5.6	4.5	2.5	0.41	0.51
	Amplitude	0.2	0.05	0.02	0.02	0.53
**CLOCK**		No rhythmic		No rhythmic		
					
					
**PER1**	Period	23.0	0.66	23.6	0.36	0.46
	Phase	10.2	2.20	15.1	0.44	0.06
	Amplitude	0.05	0.02	0.06	0.01	0.59
**PER2**	Period	25.1	0.38	23.6	0.98	0.21
	Phase	14.3	0.15	16.1	0.68	**0.04**
	Period	0.05	0.02	0.04	0.01	0.86
**CRY1**		Not rhythmic		Not rhythmic		
					
					
**CRY2**	Period	24.0	0.46	23.8	0.61	0.85
	Phase	13.8	1.10	15.6	0.61	0.19
	Amplitude	0.14	0.05	0.13	0.02	0.99
**DBP**	Period	20.8	0.30	22.8	0.55	**0.01**
	Phase	14.2	0.51	15.1	0.19	0.14
	Amplitude	0.27	0.12	0.40	0.18	0.55
**RORα**	No rhythmic			Not rhythmic		
					
					
**NR1D1**	Period	23.2	0.29	23.1	0.58	0.89
	Phase	8.9	0.74	10.4	0.82	0.20
	Amplitude	0.74	0.28	1.03	0.13	0.37
**NR1D2**	Period	24.0	0.74	23.8	0.42	0.82
	Phase	13.4	0.34	14.3	0.16	**0.04**
	Amplitude	0.32	0.10	0.35	0.05	0.78

Rhythmicity was tested using ASRER analysis at the group level.

We also compared rhythmic expression levels between young- APCs vs. older- APCs using linear mixed model. If a significant interaction was detected, this was followed by *post hoc* comparisons to determine differences in mRNA expression at specific timepoints within the rhythmic profile. Overall, CLOCK gene expression was significantly higher in older-APCs vs. young-APCs all at time points, except t = 18. PER2 gene expression was significantly higher in older-APCs vs. young-APCs at t = 42 and t = 48, with a trend towards significance at t = 24. RORα gene expression was higher in older- APCs vs. young- APCs at t = 30.

### RSV increases SIRT1 mRNA in APCs

**Figure 2:**
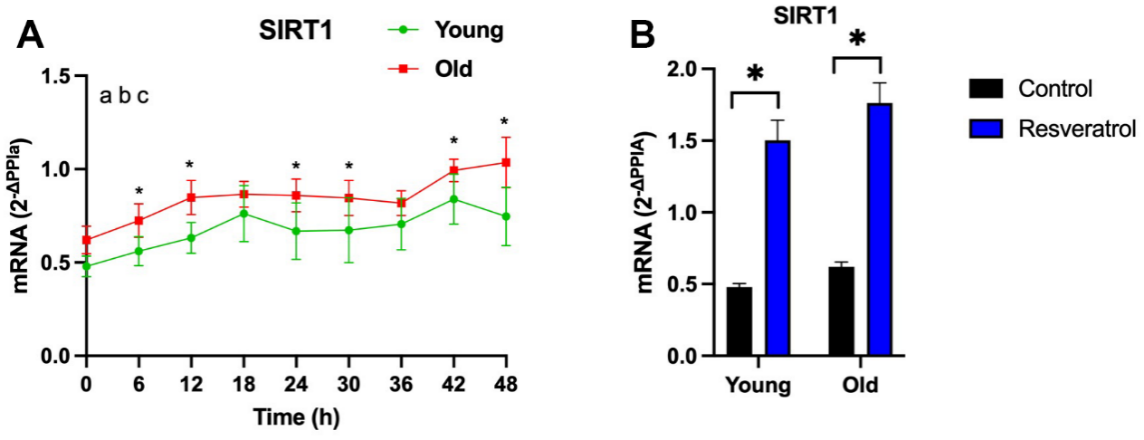
**Resveratrol treatment increased SIRT1 expression in adipose-derived progenitor cells (APCs) from young and older participants.** (**A**) SIRT1 mRNA abundance measured after completion of the vehicle control during time course full time course (every 6h for 48 h) in young-APCs (green) vs. older-APCs (red). The effect of time, age and interactions was analyzed by linear mixed model. a: *P*<0.05 effect of time; b: *P*<0.05 effect of age; c: *P*<0.05 time by age interaction. (**B**) SIRT1 mRNA abundance measured after completion of the vehicle control (black) or RSV treatment (blue) at t = 0 in both young-APCs vs. older-APCs. APCs were synchronized with 30% fetal bovine serum (FBS) for two hrs and treated with vehicle for 12 hrs. RNA was extracted from APCs, harvested every six hrs from 12 to 60 hrs post-synchronization. Abundance of transcripts was quantified by RT-PCR and normalized to Peptidylprolyl isomerase A (PPIA). Data are presented as mean ± SEM.

In order to study the effects of RSV on APCs, we first compared SIRT1 mRNA expression profiles between young vs. older-APCs at baseline (e.g. no vehicle control) using linear mixed model followed by *post hoc* comparisons. Surprisingly, SIRT1 expression was significantly higher in older-APCs at t = 6, t = 12, t = 24, t = 30, t = 42, and t = 48 compared to young-APCs (Figure 2A; *P*<0.05).

As an indicator of RSV treatment, we tested the effects of RSV on SIRT1 mRNA expression profiles in young and older-APCs using the following two conditions: 100μM RSV vs. vehicle control. 100μM RSV for 4h has been previously shown to increase clock gene expression in fibroblasts derived from young and older humans [20], which supports our rationale for using this concentration. To examine if the duration and concentration of RSV affects SIRT1 expression, we compared SIRT1 mRNA levels between RSV vs. vehicle control at t = 0. Although SIRT1 was increased in older-APCs relative to young-APCs at baseline, RSV treatment further increased SIRT1 expression in both young (by 313.2%; *P*<0.0001) and older APCs (by 283.9%; *P*<0.0001; Figure 2B).

### Effects of resveratrol on *in vitro* rhythmic clock gene expression in APCs

**Figure 3:**
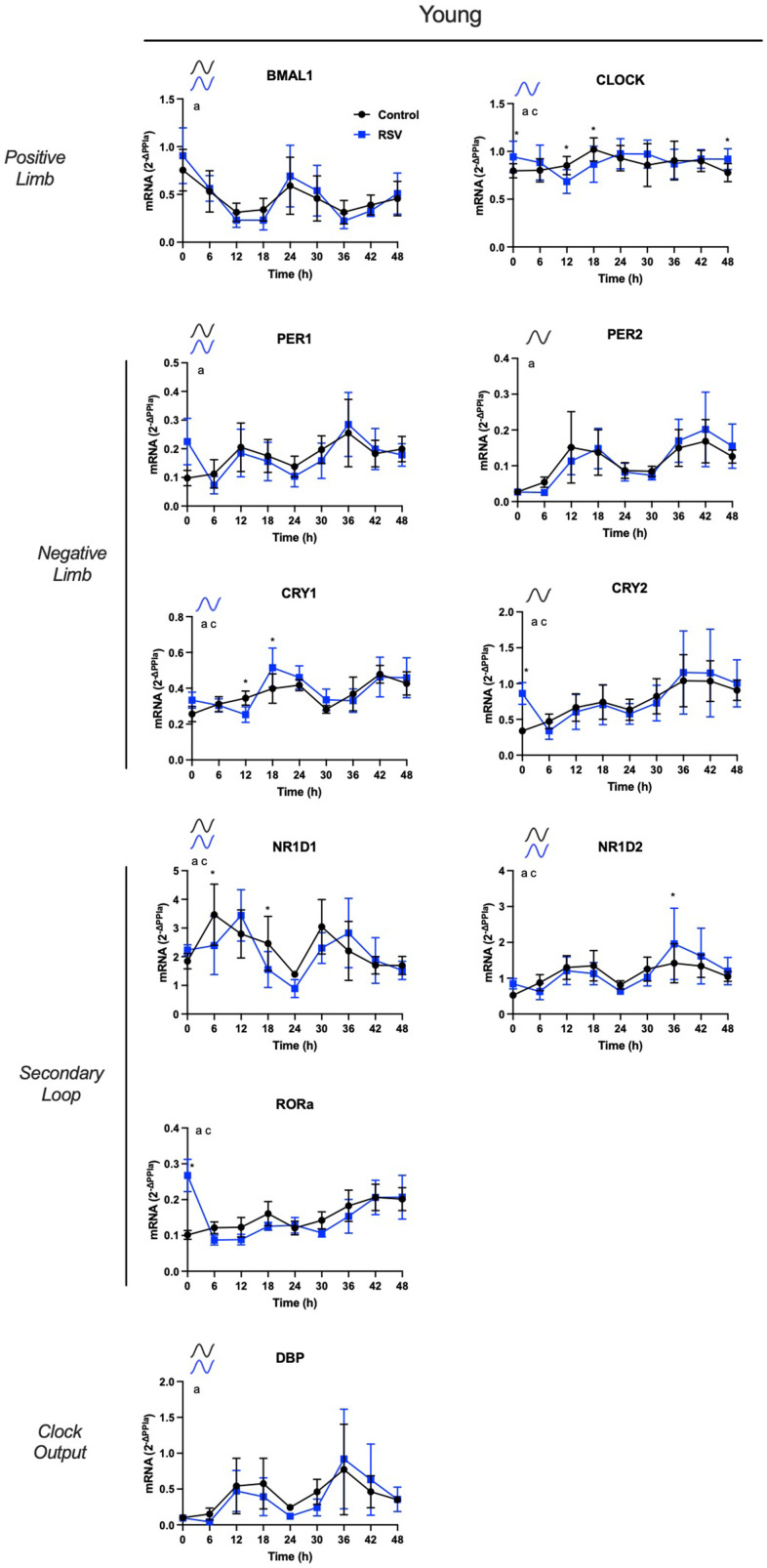
**Resveratrol increases *in vitro* rhythmic mRNA expression of core clock components in human adipose-derived progenitor cells (APCs) from young participants.** Time course analysis of *in vitro* rhythmic gene expression profiles in APCs from young after RSV treatment (blue) vs. control conditions (black). APCs were synchronized with 30% fetal bovine serum (FBS) for two hrs and treated with vehicle for 12 hrs. RNA was extracted from APCs, harvested every six hrs from 12 to 60 hrs post-synchronization. Abundance of transcripts was quantified by RT-PCR and normalized to Peptidylprolyl isomerase A (PPIA). Data are presented as mean ± SEM. When ARSER analysis detected a significant rhythm (*P*<0.05), symbols in the graphics represent oscillation in control (black), in RSV (blue), or in both groups. The effect of time, age and interactions was analyzed by linear mixed model. a: *P*<0.05 effect of time; b: *P*<0.05 effect of age; c: *P*<0.05 time by age interaction. * indicates significant differences using post-hoc comparisons. BMAL1: brain and muscle ARNT-like protein-1, CLOCK: circadian locomotor output cycles kaput; PER1/2: period 1/2, CRY1/2: cytochrome 1/2, D site of albumin promoter (albumin D-box) binding protein, RORα: RAR related orphan receptor A; NR1D1/2, nuclear receptor subfamily 1 group D member 1/2.

*Young-APCs*: We tested for significant circadian rhythmicity, using ARSER analysis, on *in vitro* rhythmic profiles in RSV vs. control. After RSV treatment, CRY1 and CLOCK gained circadian rhythmicity, PER1 and CRY2 lost circadian rhythmicity, and BMAL1, PER1, NR1D1, NR1D2, and DBP rhythmic patterns were not changed (Figure 3). Regarding rhythmic parameters, RSV resulted in longer period length for DBP expression, increased NR1D1 amplitude and phase shift, and increased NR1D2 amplitude (Table 3; all *P*< 0.05).

**Table 3:** Comparison of rhythmic parameters of *in vitro* rhythmic clock gene expression profiles in human adipose-derived progenitor cells (APCs) isolated from young participants with resveratrol (RSV) treatment vs. vehicle control condition.

**Young-APCs**						
**Gene**	**Parameter**	**Control (N=5)**		**RSV (N=5)**		**P-value**
		**Average**	**SEM**	**Average**	**SEM**	
**BMAL1**	Period	23.5	0.94	24.1	0.67	0.64
	Phase	5.6	4.5	1.3	0.49	0.36
	Amplitude	0.2	0.05	0.30	0.06	0.18
**CLOCK**	Period	Not rhythmic		24.9	0.45	--
	Phase			10.2	1.04	--
	Amplitude			0.10	0.02	--
**PER1**	Period	23.0	0.66	23.2	0.77	0.86
	Phase	10.2	2.20	15.7	1.28	0.06
	Amplitude	0.05	0.02	0.05	0.02	0.97
**PER2**	Period	25.1	0.38	Not rhythmic		--
	Phase	14.3	0.15			--
	Period	0.05	0.02			--
**CRY1**	Period	Not rhythmic		25.2	0.72	--
	Phase			22.1	0.33	--
	Period			0.11	0.01	--
**CRY2**	Period	24.0	0.46	Not rhythmic		--
	Phase	13.8	1.10			--
	Amplitude	0.14	0.05			--
**DBP**	Period	20.8	0.30	22.7	0.53	**0.01**
	Phase	14.2	0.51	14.6	0.34	0.57
	Amplitude	0.27	0.12	0.29	0.10	0.89
**RORα**	Period	Not rhythmic		Not rhythmic		--
	Phase					--
	Period					--
**NR1D1**	Period	23.2	0.29	23.4	0.55	0.68
	Phase	8.9	0.74	10.9	0.51	**<0.005**
	Amplitude	0.74	0.28	0.84	0.12	**0.006**
**NR1D2**	Period	24.0	0.74	23.8	1.16	0.88
	Phase	13.4	0.34	13.9	1.29	0.38
	Amplitude	0.32	0.10	0.35	0.10	**0.02**

Rhythmicity was tested using ASRER analysis at the group level.

We then compared rhythmic gene expression levels of these same conditions using linear mixed model followed by *posthoc* comparisons. RSV treatment increased mRNA expression of CLOCK (at t = 0, t = 12, t =18 and t = 48), CRY1 (at t = 12, t = 18), CRY2 (at t = 0), NR1D1 (at t = 6 and t = 18), NR1D2 (at t = 36), and RORα (at t=0).

**Figure 4:**
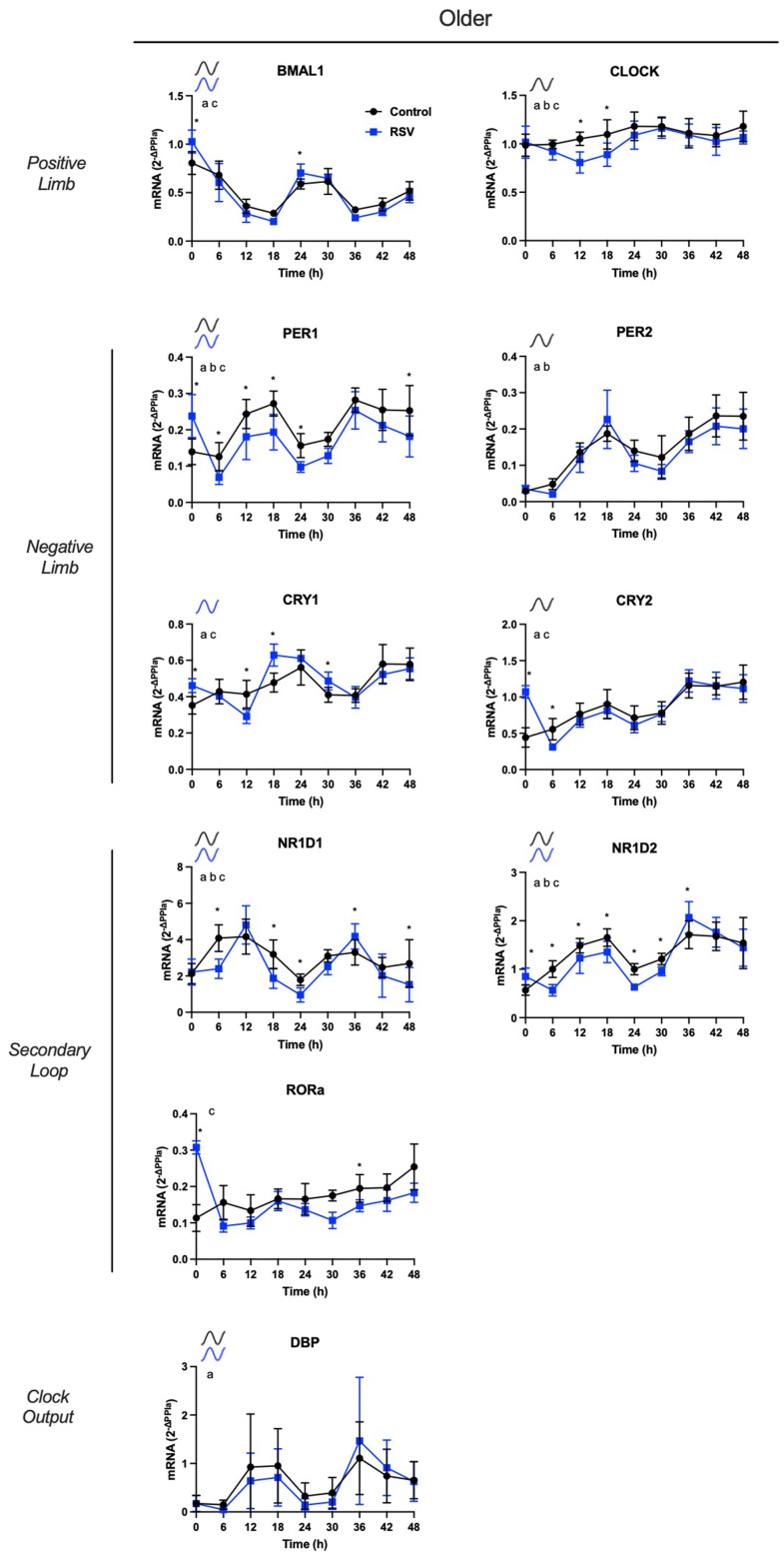
**Resveratrol increases *in vitro* rhythmic gene mRNA expression of core clock components in human adipose-derived progenitor cells (APCs) from older participants.** Time course analysis of the *in vitro* rhythmic gene expression profiles in APCs from older participants after RSV treatment (blue) vs. control conditions (black). APCs were synchronized with 30% fetal bovine serum (FBS) for two hrs and treated with vehicle for 12 hrs. RNA was extracted from APCs, harvested every six hrs from 12 to 60 hrs post-synchronization. Abundance of transcripts was quantified by RT-PCR and normalized to Peptidylprolyl isomerase A (PPIA). Data are presented as mean ± SEM. When ARSER analysis detected a significant rhythm (*P*<0.05), symbols in the graphics represent oscillation in control (black), in RSV (blue), or in both groups. The effect of time, age and interactions was analyzed by linear mixed model. a: *P*<0.05 effect of time; b: *P*<0.05 effect of age; c: *P*<0.05 time by age interaction. * indicates significant differences using post-hoc comparisons. BMAL1: brain and muscle ARNT-like protein-1, CLOCK: circadian locomotor output cycles kaput; PER1/2: period 1/2, CRY1/2: cytochrome 1/2, D site of albumin promoter (albumin D-box) binding protein, RORα: RAR related orphan receptor A; NR1D1/2, nuclear receptor subfamily 1 group D member 1/2.

*Older-APCs*: RSV treatment had the same effects on circadian rhythmicity of core clock components, as was observed in young-APCs. More specifically, after RSV treatment, CRY1 and CLOCK gained circadian rhythmicity, PER1 and CRY2 lost circadian rhythmicity, and BMAL1, PER1, NR1D1, NR1D2, and DBP rhythmic patterns were not changed (Figure 4). Regarding rhythmic parameters, RSV treatment resulted in PER1 phase shift, increased NR1D1 amplitude, and NR1D2 phase shift (Table 4; all *P*<0.05).

**Table 4:** Comparison of rhythmic parameters from *in vitro* rhythmic clock gene expression profiles in human adipose-derived progenitor cells (APCs) isolated from older participants with resveratrol (RSV) treatment vs. vehicle control condition.

	**Older-APCs**					
**Gene**	**Parameter**	**Control (N=5)**		**RSV (N=5)**		**P-value**
		**Average**	**SEM**	**Average**	**SEM**	
**BMAL1**	Period	24.1	0.21	25.4	0.77	0.15
	Phase	2.5	0.41	6.5	5.05	0.33
	Amplitude	0.02	0.02	0.3	0.01	0.75
**CLOCK**	Period	Not rhythmic		24.5	0.45	--
	Phase			3.2	1.04	--
	Amplitude			0.10	0.02	--
**PER1**	Period	23.6	0.36	23.0	0.58	0.42
	Phase	15.1	0.44	17.1	0.65	**0.04**
	Amplitude	0.06	0.01	0.06	0.01	0.99
**PER2**	Period	23.6	0.98	Not rhythmic		--
	Phase	16.1	0.68			--
	Period	0.04	0.01			--
**CRY1**	Period	Not rhythmic		25.2	0.72	--
	Phase			22.1	0.33	--
	Period			0.11	0.01	--
**CRY2**	Period	23.8	0.61	Not rhythmic		--
	Phase	15.6	0.61			--
	Amplitude	0.13	0.02			--
**DBP**	Period	22.8	0.55	21.7	0.83	0.33
	Phase	15.1	0.19	16.1	0.73	0.23
	Amplitude	0.40	0.18	0.47	0.22	0.83
**RORα**	Period	Not rhythmic		Not rhythmic		--
	Phase					--
	Period					--
**NR1D1**	Period	23.1	0.58	25.1	1.27	0.20
	Phase	10.4	0.82	11.2	0.68	0.74
	Amplitude	1.03	0.13	1.49	0.19	**0.03**
**NR1D2**	Period	23.8	0.42	22.0	0.92	0.12
	Phase	14.3	0.16	15.8	0.48	**0.02**
	Amplitude	0.35	0.05	0.45	0.04	0.22

Rhythmicity was tested using ASRER analysis at the group level.

We then compared rhythmic gene expression levels of these same conditions using linear mixed model followed by posthoc comparisons. RSV treatment increased mRNA expression of BMAL1 (at t = 0 and t = 24), CLOCK (t = 12 and t =18), PER1 (at t = 0, t = 6, t = 12, t = 18, t = 24, and t = 48), CRY1 (at t = 0, t = 12, t = 18, and t = 30), CRY2 (t = 0 and t = 6), NR1D1 (at t = 6, t = 18, t = 24, t = 36, and t = 48), NR1D2 (at t = 0, t = 6, t = 12, t = 18, t = 24, t = 30, and t = 36), and RORα (at t = 0 and t = 36).

## DISCUSSION

The primary finding of this study was that preadipocytes derived from subcutaneous adipose tissue biopsies from both young and older female participants exhibited significant circadian rhythmicity in the gene expression patterns of the molecular machinery of the core clock mechanism, including the positive limb (BMAL1), negative limb (PER1, PER2, CRY2), and secondary loop (NR1D1, NR1D2), as well as clock output gene DBP. CLOCK, CRY1, and RORα were not significantly rhythmic. These experiments were conducted *in vitro,* giving insight into the function of the core clock independent of influence of the *in vivo* environment. Thus, our observations suggest that the oscillatory function of the cell-autonomous core clock mechanism in preadipocytes is largely preserved at this stage of the aging process in female participants. This finding may be related to the model of aging used in the study, which consisted of preadipocytes from a female participant cohort with the mean age of 70.6 yrs without overt metabolic disease. Previous studies demonstrate slowed proliferation rates in preadipocytes derived from older adults of similar ages (average ages of 71 and 66 yrs) compared to young adults, supporting that some age-related changes in adipose tissue occur in the 6^th^ and 7^th^ decade of life that are detectable *in vitro* [25, 26]. In light of this early work, perhaps our current findings of robust circadian oscillations in preadipocytes derived from older adults aligns with the paradigm that healthy aging is associated with robust circadian rhythms [24].

Although the circadian rhythmicity patterns measured *in vitro* were consistent between young and older preadipocytes, we observed increased expression levels of CLOCK, PER2, NR1D1 in older-APCs compared to young-APCs. Differences in gene expression levels across ages have been previously reported in other tissues [27, 28]. For example, human colon tissue from participant donors over 74 yrs exhibits increased CLOCK, PER2, and BMAL1 expression, compared to colon tissue from younger participants [27]. Similarly, rat liver tissue from middle-aged animals exhibits increased NR1D1 and NR1D2 expression, compared to rat liver tissue from young [28]. Further, age associated changes in core clock expression are tissue-specific, and while speculative, these changes may be part of beneficial or compensatory adaptations to the aging process [29]. Thus, our findings are consistent with the hypothesis that functional clocks are a characteristic feature of healthy aging, which we extend to the context of preadipocytes derived from healthy older adults, which, to the best of our knowledge, has not been previously investigated.

Another finding of this study was that administration of exogenous RSV increased the expression of core clock genes in preadipocytes derived from both young and older female participants, which suggests that the ability of the core clock to sense and respond to stimuli is retained in this model of aging. Our findings of increased clock gene expression after RSV agree with previous work in rat fibroblasts [30], yet we extend these questions within the context of aging which has not been done previously. There were some differences in the effects of RSV on rhythmic patterns and expression levels between young and older preadipocytes, which is consistent with previous reports in lung fibroblasts from young and older adults [20]. In both young and older preadipocytes, the RSV-induced alterations in rhythmic patterns of gene expression are most likely explained by a transient effect of the RSV treatment that dissipated over the duration of the 48h assessment period. For example, the effect of RSV on young and older preadipocytes was most evident as increased gene expression at t = 0, a timepoint that corresponds to the immediate completion of the 12hr RSV treatment. The later time points of the rhythmic gene expression profile were also influenced by RSV, however to a lesser extent than t = 0. Overall, our findings that RSV increases expression of core clock components in young and older preadipocytes are in agreement with previous studies indicating that RSV interacts with the core clock [20, 30] and highlight the novel context of human preadipocytes.

RSV treatment increased SIRT1 expression in both young and older preadipocytes, which is consistent with previous work in other cell and tissue types [20, 21]. One surprising finding is that we observed higher SIRT1 mRNA expression in older preadipocytes compared to young, at baseline (or, vehicle control conditions) and the explanation for this is unclear. Older adults may have increased SIRT1 levels due to declines in NAD+ consumption by other metabolic pathways as a result of declining energetic demands [31, 32]; or this finding may reflect lower electron transport chain activity, particularly at Complex I [33]. Interestingly, these increased SIRT1 expression levels were accompanied by robust circadian oscillations, suggesting that our current model of aging may represent a relatively healthy phenotype.

There are a few limitations that warrant mention. Our participant cohort consisted of 10 females participants; thus, generalizing findings back to the broader population including males is limited. Sex differences in circadian functions have been previously reported, [34] so it is plausible that sex differences are also present in the core clock of human adipose tissue and this remains an open area of investigation. Another important limitation is the relatively low sampling frequency used to determine rhythmic patterns of mRNA expression of core clock. We harvested APCs every 6h for 48h, resulting in 4 timepoints per circadian cycle. This temporal resolution is limited and reduces the statistical ability to detect a significant circadian rhythm. Alternative methods, such as luciferase report assays, survey temporal changes at a higher frequency (every 15 minutes) and would provide more detailed information about the rhythmic patterns. Phase shifts in genes of older preadipocytes were observed compared to young, as well as effects of RSV on phase shifts and rhythmic period on some genes; yet these findings should be interpreted cautiously due to limited temporal resolution. We selected experimental conditions based on prior work [2, 20] (e.g. synchronization procedures and RSV concentrations), yet the extent to which our findings translate to experimental conditions beyond what was tested here is not known. Despite these limitations, this current work contributes to the aging field because it is the first study to interrogate age associated changes in core clock mRNA expression in human preadipocytes.

This study reflects transdisciplinary efforts to link aging and circadian biology in human-derived tissues, which is an understudied area of biology. Consistent patterns of circadian rhythmicity of core clock components were observed in young and older preadipocytes derived from female participants, suggesting that the oscillatory function of the cell-autonomous core clock in preadipocytes is preserved from early adulthood (~20 yrs) to early aging (~70 yrs). The finding of preserved clock function during aging is consistent with other work in the field; previous approaches using preclinical models report that while the core clock works during aging, clock output is impacted in a tissue-specific manner [2]. To further investigate age associated changes in circadian function, future studies could explore experimental models of healthy aging versus non-healthy aging. Also, models of aging beyond 70 years old, as well as addressing tissue-specific changes in circadian function across the lifespan would provide a more comprehensive advance of the field. We also observed that the core clock in young and older preadipocytes responds to exogenous RSV administration, potentially suggesting that RSV may be a useful tool for promoting longevity and health by targeting the core clock in adipose tissue.

## MATERIALS AND METHODS

### Participant donors

10 female participants completed the BioAge study at the Translational Research Institute, AdventHealth (AH TRI) in Orlando, Florida, The United States of America. Five participants were classified as older (aged 65-79) and five as young (aged 20-25). All included participants had a BMI ≤ 40 kg/m^2^, were weight stable (±5 kg) for the last three months prior to the screening visit and were sedentary (<20 min of activity, 3x/week). Participants with a medical history of diabetes, coronary heart disease, stroke, chronic renal failure, chronic hepatic disease, autoimmune disease or gastrointestinal disease requiring medication, use of insulin or any other glucoregulatory drugs were excluded. Participants were not taking anti-inflammatory (within two days prior to biopsy) or antiplatelet medications (within seven days prior to biopsy), were not pregnant or less than 9 months postpartum. Other exclusion criteria included: drug or alcohol abuse (≥3 drinks per day) within the last five years, usage of oral contraceptives (<3 months on a stable regime) (with the exception of hormone replacement therapy), usage of tobacco or nicotine-containing products (within the past three months).

### Adipose tissue biopsies

Adipose tissue biopsies of the abdominal subcutaneous region were performed under local anesthesia [35]. Adipose biopsies were taken in the overnight fasted state between 8 and 10 AM. The scWAT was digested with collagenase (type 1, 1 mg/2ml in HBSS) for 30-60 min at 37° C with shaking (100rpm), as described previously [36]. The mixture was passed through a 250-micron mesh, centrifuged at 500g for five minutes and floating adipocytes were discarded. Cell pellets were treated with erythrocyte lysis buffer (BioLegend, Cat: 420301) for five to 10 minutes at room temperature followed by centrifugation at 500g for five minutes. After discarding the supernatant, cells were resuspended in proliferation media (PM) (Gibco α-Minimum Essential Media (αMEM), 10% Fetal Bovine Serum (FBS) and 1% Penicillin-Streptomycin), then plated and cultured in an incubator at 37° C at 5% carbon dioxide. Media was replaced after four hours to remove blood cells. APCs continue to grow with PM changes every other day until they reached ~80% confluency, then they were frozen in freezing media (10% FBS, 10% DMSO in αMEM) and stored in dewars containing liquid nitrogen.

### APC cell culture procedures

APCs frozen cryovials were quickly thawed by directly placing them in a 37° C water bath, then centrifuged at 500g for five min to remove freezing media. APCs were plated in a T75 flask with 10mL of PM. The PM was replaced every two or three days until cells were ~80% confluent. Once confluent, cells were lifted by trypsin and cell number was determined by using trypan blue exclusion and an automatic cell counter (Countess II FL). The APCs were plated in a 12-well plate with αMEM and 5% FBS at a density of 50,000 cells per well. All APCs were passage 3-5 at the time of the experiment. *In vitro* synchronization for APCs was initiated 24 hours after plating.

### In vitro synchronization

After 24h, cells were 70% confluent. Subsequently, cells were synchronized by adding media containing 30% FBS for 2h, as previously performed [8]. Subsequently, the αMEM containing with 2% FBS was added for 12h. Cells were harvested every 6h for a period of 48h.

### Resveratrol treatment

RSV (3,5,40-trihydroxystilbene, purity ≥99%) was purchased from Sigma-Aldrich (R5010). 100mM RSV stock solution in 100% ethanol was made. APCs were synchronized as described above for 2h. Cells were treated with 100μM RSV or ethanol (vehicle) for 12h, and then cells were harvested every 6h for a period of 48h [7, 20].

### RNA isolation and real-time quantitative reverse transcriptase-PCR

To isolate RNA from APCs, we used the RNeasy Lipid Tissue Mini kit (Qiagen, Cat: NC9307831). 200μL of RLT buffer per well was used. 2 wells of 12 well plates were combined per treatment. Harvested cells were immediately frozen at -80° C. RNA was isolated according to the protocol, and quality and quantity were assessed by using the Nanodrop spectrophotometer (Thermo Fisher Scientific, Waltham, MA, USA). The PCR reagents were 2.5μL of 4X TaqMan Fast Virus Master Mix (Applied Biosystems, Cat: 4444434), 0.5μL of 20X primer and 10ng of RNA for a total reaction of 10μL. The initial step of PCR was five minutes at 50° C of reverse transcription, followed by a 20-second hold at 95° C for inactivation/initial denaturation. Followed by 40 cycles of PCR amplification for 15 seconds at 95° C and 60 seconds at 60° C. PCR reactions were performed in triplicate in 384-wells plates. The housekeeping gene peptidylprolyl isomerase A (PPIA) (Hs_01565699_g1) was used to normalize core clock gene expressions calculated by 2(^–ΔCt^) method. PPIA was previously identified as a suitable reference gene in scWAT of mice fed during the resting or active phase, thus, this gene is optimal for studies of clock genes [37]. Pre-designed TaqMan gene expression assays from Applied Biosystems were: CLOCK (Hs00231857_m1), BMAL1 (Hs00154147_m1), PER1 (Hs00242988_m1), PER2 (Hs00256143_m1), CRY1 (Hs00172734_m1), CRY2 (Hs00323654_m1) and SIRT1 (Hs01009006_m1). The resulting transcript abundance was measured by a Real-Time Polymerase Chain Reaction (RT-PCR) (Applied Biosystems, Waltham, MA, USA).

### Statistics

Statistical analyses were performed using SAS (9.4) or Prism (v9.3.0, GraphPad Software Inc.). Results were presented as mean ± standard error of the mean (SEM) unless stated otherwise. Two-tailed P-values ≤ 0.05 were considered statistically significant. The Shapiro-Wilk W-test was used to test the normality of the data. For between group comparisons a two-sample t-test or a Fisher’s exact test was performed. To analyze the differences between the age groups over time and the differences between the control and resveratrol treated group over time for the young and older participants, a linear mixed model for repeated measures over time was used. The final mixed model included group, time and their interactions. The rhythmicity of gene expression was analyzed using ARSER method of R MetaCycle package [38].
